# Supplementary material for: Chimeric Claudins: A New Tool to Study Tight Junction Structure and Function
Source: Int J Mol Sci. 2021 May 6;22(9):4947. doi: 10.3390/ijms22094947 (PMC8124314; doi:10.3390/ijms22094947)

Supplementary Information

Supplementary Figure S1. pET28-MBP (Kanamycin resistance)

```
        10         20         30         40         50         60
MGKIEEGKLV IWINGDKGYN GLAEVGKKFE KDTGIKVTVE HPDKLEEKFP QVAATGDGPD

        70         80         90        100        110        120
IIFWAHDRFG GYAQSGLLAE ITPDKAFQDK LYPFTWDAVR YNGKLIAYPI AVEALSLIYN

       130        140        150        160        170        180
KDLLPNPPKT WEEIPALDKE LKAKGKSALM FNLQEPYFTW PLIAADGGYA FKYENGKYDI

       190        200        210        220        230        240
KDVGVDNAGA KAGLTFLVDL IKNKHMNADT DYSIAEAAFN KGETAMTING PWAWSNIDTS

       250        260        270        280        290        300
KVNYGVTVLP TFKGQPSKPF VGVLSAGINA ASPNKELAKE FLENYLLTDE GLEAVNKDKP

       310        320        330        340        350        360
LGAVALKSYE EELAKDPRIA ATMENAQKGE IMPNIPQMSA FWYAVRTAVI NAASGRQTVD

       370
EALKDAQTNA AAHM -INSERT-LEHHHHHH
```

Number of amino acids: 374. **Molecular weight:** 40992.61

Sites for NdeI (**HM** amino acids) and XhoI (**LE** amino acids) are present in the plasmid.

```
        10         20         30         40         50         60
MGKIEEGKLV IWINGDKGYN GLAEVGKKFE KDTGIKVTVE HPDKLEEKFP QVAATGDGPD

        70         80         90        100        110        120
IIFWAHDRFG GYAQSGLLAE ITPDKAFQDK LYPFTWDAVR YNGKLIAYPI AVEALSLIYN

       130        140        150        160        170        180
KDLLPNPPKT WEEIPALDKE LKAKGKSALM FNLQEPYFTW PLIAADGGYA FKYENGKYDI

       190        200        210        220        230        240
KDVGVDNAGA KAGLTFLVDL IKNKHMNADT DYSIAEAAFN KGETAMTING PWAWSNIDTS

       250        260        270        280        290        300
KVNYGVTVLP TFKGQPSKPF VGVLSAGINA ASPNKELAKE FLENYLLTDE GLEAVNKDKP

       310        320        330        340        350        360
LGAVALKSYE EELAKDPRIA ATMENAQKGE IMPNIPQMSA FWYAVRTAVI NAASGRQTVD

       370        380        390        400        410        420
EALKDAQTNA AAHMMYGKLN DLLEDLQEVL KHVNQHWQGI VSTALPQWRI YSYAGDNIVT

       430        440        450        460        470        480
AQAMYEGLWM SCVSQSTGQI QCKVFDSLLN LSSTLQATRA LMVVGNMNKV DHHLQNVIED

       490        500        510        520        530        540
IHDFMQGGGS GGKLQEMMKE FQQVLDEIKQ QLQGILVATA WYGNRIVQEF YDPMTPVNAR

       550        560        570        580
YEFGQALFTG WAGSLHNVHE NIKEIFHHLE ELVHRLE HHHHHH
```

Supplementary Figure S2. Amino acid sequence of Chimeric proteins. All proteins are fusion at the N-terminus with MBP and have a 6xHis. Red amino acids belong to 2jua, purple amino acids belong to claudins and other proteins of interest.

>CLDN1 chimera 2jua (50P)
MYGKLNDLLEDLQEVLKHVNQHWQGIVSTALPQWRIYSYAGDNIVTAQAMYEGLWM SCVSQSTGQIQCKVFDSLLNLSSTLQATRALMVVGNMNKVDHHLQNVIEDIHDFMQG GGSGGKLQEMMKEFQQVLDEIKQQLQGILVATAWYGNRIVQEFYDPMTPVNARYEF GQALFTGWAGSLHNVHENIKEIFHHLEELVHR

>CLDN1 chimera 2jua (40P)
MYGKLNDLLEDLQEVLKHVNQHWQGALPQWRIYSYAGDNIVTAQAMYEGLWMSCV SQSTGQIQCKVFDSLLNLSSTLQATRALNMNKVDHHLQNVIEDIHDFMQGGGSGGKL QEMMKEFQQVLDEIKQQLQGTAWYGNRIVQEFYDPMTPVNARYEFGQALFGSLHNV HENIKEIFHHLEELVHR

>CLDN1 chimera 2jua (30P)
MYGKLNDLLEDLQEVLKHVNQHWQGWRIYSYAGDNIVTAQAMYEGLWMSCVSQST GQIQCKVFDSLLNLSSTLQANMNKVDHHLQNVIEDIHDFMQGGGSGGKLQEMMKEF QQVLDEIKQQLQGGNRIVQEFYDPMTPVNARYEFGGSLHNVHENIKEIFHHLEELVHR

>CLDN1 chimera 2jua (20P)
MYGKLNDLLEDLQEVLKHVNQHWQGWRIYSYAGDNIVTAQAMYEGLWMSCVSQST GQIQCKVFDSNMNKVDHHLQNVIEDIHDFMQGGGSGGKLQEMMKEFQQVLDEIKQQ LQGYDPMTPVNARYEFGGSLHNVHENIKEIFHHLEELVHR

>CLDN1 chimera 2jua (30PΔECL1)
MYGKLNDLLEDLQEVLKHVNQHWQGGQKNMNKVDHHLQNVIEDIHDFMQGGGSGG KLQEMMKEFQQVLDEIKQQLQGGNRIVQEFYDPMTPVNARYEFGGSLHNVHENIKEI FHHLEELVHR

>CLDN1 chimera 2jua (30PΔECL2)
MYGKLNDLLEDLQEVLKHVNQHWQGWRIYSYAGDNIVTAQAMYEGLWMSCVSQST GQIQCKVFDSLLNLSSTLQANMNKVDHHLQNVIEDIHDFMQGGGSGGKLQEMMKEF QQVLDEIKQQLQGGDNSLHNVHENIKEIFHHLEELVHR

>CLDN2 chimera 2jua (50P)
MYGKLNDLLEDLQEVLKHVNQHWQGVAMLLPSWKTSSYVGASIVTAVGFSKGLWM ECATHSTGITQCDIYSTLLGLPADIQAAQAMMVTSSNMNKVDHHLQNVIEDIHDFMQG GGSGGKLQEMMKEFQQVLDEIKQQLQGLGFIPVAWNL HGILRDFYSPLVPDSMKFEIGEALYLGIIGSLHNVHENIKEIFHHLEELVHR

>CLDN3 chimera 2jua (50P)

MYGKLNDLLEDLQEVLKHVNQHWQGIVCCALPMWRVSAFIGSNIITSQNIWEGLWMN CVVQSTGQMQCKVYDSLLALPQDLQAARALIVVANMNKVDHHLQNVIEDIHDFMQG GGSGG**KLQEMMKEFQQVLDEIKQQLQGTLVPVSWSANTIIRDFYNPVVPEAQKREM GAGLYVGWAGSLHNVHENIKEIFHHLEELVHR**

>OCLN chimera 2jua (50P)

MYGKLNDLLEDLQEVLKHVNQHWQGASTLAWDRGYGTSLLGGSVGYPYGGSGFG SYGSGYGYGYGYGYGYGGYTDPRAAKGFMLANMNKVDHHLQNVIEDIHDFMQGGG SGGKLQEMMKEFQQVLDEIKQQLQGATIVYIMGVNPTAQSSGSLYGSQIYALCNQFY TPAATGLYVDQYLYHYCVVDPQEAIAIVLGGSLHNVHENIKEIFHHLEELVHR

>C-terminus CPE (amino acids 194 to 319)
MENLYFQSGSGYPYDVPDYAGSGDIEKEILDLAAATERLNLTDALNSNPAGNLYDWRS SNSYPWTQKLNLHLTITATGQKYRILASKIVDFNIYSNNFNNLVKLEQSLGDGVKDHYV DISLDAGQYVLVMKANSSYSGNYPYSILFQKF

>C- terminus CPE (m19)

MENLYFQSGSGYPYDVPDYAGSGDIEKEILDLAAATERLNLTDALNSNPAGNLYDWRS SNSYPWTQKLNLHLTITATGQKYRILASKIVDFNIYSNNFNNLVKLEQSLGDGVKDHYV DISLDAGQYVLVMKANapYrGhYPYhILFQKF

*** Amino acids highlighted in yellow are mutated in CPE(m19)

>Zebrafish CLDN11A chimera 2jua

MYGKLNDLLEDLQEVLKHVNQHWQGVATATNEWVFTCKYQMNTCRKMDELEAKGL WADCVISTALYHCITLTQILELPAYIQTSRALMVTASNMNKVDHHLQNVIEDIHDFMQG GGSGGKLQEMMKEFQQVLDEIKQQLQGCGIVSTVWFPIGAHHEKGLMSFGFSLYSG WVGSLHNVHENIKEIFHHLEELVHR

Supplementary Figure S3.

Ligand preparation for SPR experiments

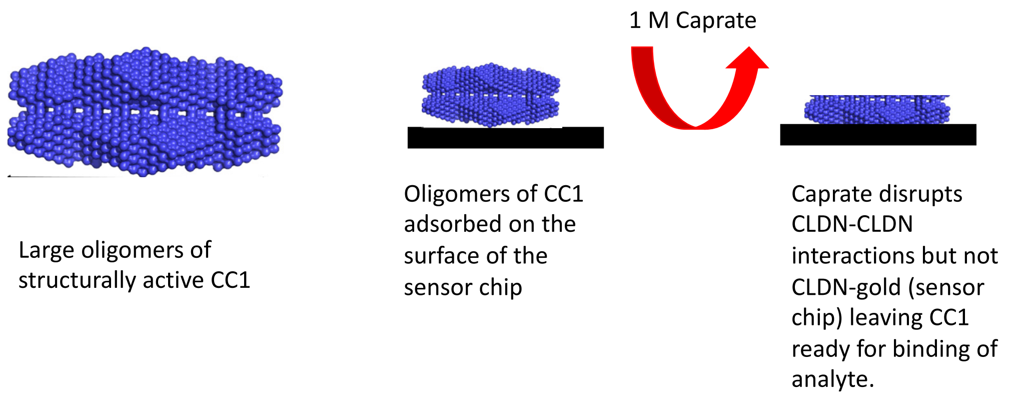

Supplement: Supplementary file 1 [file ijms-22-04947-s001.zip › ijms-1168206-supplementary.pdf]
